# Draft Genome Sequence of *Achromobacter* sp. Strain DMS1, Capable of Degrading Polyaromatic Hydrocarbons Isolated from the Industrially Perturbed Environment of Amlakhadi Canal, India

**DOI:** 10.1128/genomeA.01264-15

**Published:** 2015-10-22

**Authors:** Shivani Amin, Binal Shah, Kunal Jain, Anand Patel, Namrata Patel, Chaitanya G. Joshi, Datta Madamwar

**Affiliations:** a Environmental Genomics and Proteomics Lab, BRD School of Biosciences, Satellite Campus, Sardar Patel University, Bakrol, Gujarat, India; b Department of Animal Biotechnology, College of Veterinary Science and Animal Husbandry, Anand Agricultural University, Anand, Gujarat, India

## Abstract

Here, we report the draft genome sequence of *Achromobacter* sp. strain DMS1, which is 4.9 Mbp and has 3,727 coding sequences (CDSs), and is capable of degrading xenobiotic compounds and harboring genes for aromatic hydrocarbon metabolism. Its genome will unravel the basic mechanism involved in bioremediation of anthropogens.

## GENOME ANNOUNCEMENT

A broad array of monoaromatic and polyaromatic compounds is released into an open environment through natural catastrophes, hydrocarbon seeps, and anthropogenic origin (*viz*. crude oil drilling, incomplete combustion of petroleum products, disproportionate use of synthetic compounds of aromatic nature, etc.). The benzyl ring is the basic unit of polyaromatic compounds and is the second most abundant chemical structure found in the biosphere, and its thermodynamic properties increases their persistence in nature (1). However, inhabitant microbes have evolved the basic mechanism of metabolizing such xenobiotic compounds.

*Achromobacter* sp. DMS1, a mesophilic, motile, polycyclic aromatic hydrocarbon (PAH)-degrading bacterium was isolated from sediments of long term industrially perturbed ecosystem of Amlakhadi canal, Ankleshwar Industrial Estate, Ankleshwar, Gujarat, India. Its genome was sequenced to understand how the basic mechanism(s) evolved due to adaptation toward compounds of xenobiotic origin and to mine genes relevant to bioremediation.

The whole genome was sequenced by both shotgun and mate-paired library sequencing on an Ion Torrent sequencer using a 316 chip, with 200 bp chemistry. The shotgun sequencing produced 1,822,780 reads, 1,807,992 reads in mate-paired libraries.

The reads were assembled using GS *de novo* Assembler v2.6, CLC genomics workbench software (CLC Bio-Qiagen, Aarhus, Denmark), MIRA (2), and SPAdes (3) assemblers. All four assemblies were integrated using a CISA (4) and further assembled by SSPACE (5). The draft genome was assembled into 356 scaffolds of 4,941,329 bp with 67.4% G+C content.

The draft genome was annotated using the Rapid Annotations using Subsystems Technology (RAST) server (6) and the NCBI Prokaryotic Genome Annotation Pipeline (PGAAP) (http://www.ncbi.nlm.nih.gov/genome/annotation_prok). By using both tools, we have predicted that the draft genome of *Achromobacter* sp. DMS1 carries 3,727 coding sequences (CDSs) with 678 subsystems, 63 structural RNAs encoding genes (8 rRNA and 55 tRNA), and 1 gene for noncoding RNA (ncRNA). Among the 678 subsystems, besides genes related to normal metabolism and housekeeping functions, genes related to stress response, virulence, disease and defense, metabolism of aromatic compounds, motility, sulfur and nitrogen metabolism, beta-lactamase activity, multidrug transporters, and formyltetrahydrofolate deformylase were mapped.

The annotation revealed that genome of strain DMS1 contains a large number of genes responsible for the xenobiotic degradation. It included FAD-dependent oxidoreductase, arsenate, nitrate, nitrite and sulfite reductase, cobalt transporter, catechol-1,2-dioxygenase, aromatic-ring opening dioxygenase (ligB), and ammonia monooxygenase. Genes responsible for protocatechuate degradation, 3-hydroxycinnamate degradation, shikimate dehydrogenase, tartrate dehydrogenase, and NAD(P)H dehydrogenase were also annotated. The complete pathway for benzonitrile, benzamide, benzoate, and catechol degradation was mapped. The genome annotation revealed the presence of genes for chemotaxis (cheY, cheD, cheW, cheA) which signifies the role of the strain DSM1 in xenobiosis (7).

We are now exploring the genome of *Achromobacter* sp. DMS1 to understand the mechanism of resistance toward xenobiotic compounds and to isolate the gene(s), biochemical pathways, metabolites involved in xenobiotic degradation.

### Nucleotide sequence accession numbers. 

This whole-genome shotgun (WGS) project has been deposited at DDBJ/EMBL/GenBank under the accession number LHSJ00000000. The version described in this paper is version LHSJ01000000.
